# The physiological roles of tau and Aβ: implications for Alzheimer’s disease pathology and therapeutics

**DOI:** 10.1007/s00401-020-02196-w

**Published:** 2020-07-29

**Authors:** Sarah A. Kent, Tara L. Spires-Jones, Claire S. Durrant

**Affiliations:** 1 Translational Neuroscience PhD Programme, Centre for Discovery Brain Sciences and the UK Dementia Research Institute, The University of Edinburgh, 1 George Square, Edinburgh, EH8 9JZ Scotland, UK; 2 Centre for Discovery Brain Sciences and the UK Dementia Research Institute, The University of Edinburgh, 1 George Square, Edinburgh, EH8 9JZ Scotland, UK

**Keywords:** Synapse, Myelination, Vasculature, Memory, Therapeutics, Microtubule dynamics

## Abstract

Tau and amyloid beta (Aβ) are the prime suspects for driving pathology in Alzheimer’s disease (AD) and, as such, have become the focus of therapeutic development. Recent research, however, shows that these proteins have been highly conserved throughout evolution and may have crucial, physiological roles. Such functions may be lost during AD progression or be unintentionally disrupted by tau- or Aβ-targeting therapies. Tau has been revealed to be more than a simple stabiliser of microtubules, reported to play a role in a range of biological processes including myelination, glucose metabolism, axonal transport, microtubule dynamics, iron homeostasis, neurogenesis, motor function, learning and memory, neuronal excitability, and DNA protection. Aβ is similarly multifunctional, and is proposed to regulate learning and memory, angiogenesis, neurogenesis, repair leaks in the blood–brain barrier, promote recovery from injury, and act as an antimicrobial peptide and tumour suppressor. This review will discuss potential physiological roles of tau and Aβ, highlighting how changes to these functions may contribute to pathology, as well as the implications for therapeutic development. We propose that a balanced consideration of both the physiological and pathological roles of tau and Aβ will be essential for the design of safe and effective therapeutics.

## Introduction

Alzheimer’s disease (AD) is a terminal neurodegenerative disorder associated with severe progressive dementia [155]. The disease is characterised by key neuropathological hallmarks of chronic inflammation, synapse loss, neuronal death and the diagnostic accumulation of insoluble protein aggregates, intracellular neurofibrillary tangles (NFTs), and extracellular amyloid plaques [28, 155, 258]. The disease begins as a primary disorder of short-term memory, learning, and spatial navigation, due to early degeneration of the temporal lobe [174]. In the end stages, however, the spread of pathology throughout the brain results in multimodal deficits, including loss of verbal and motor control [174]. Unfortunately, AD is a common disorder, affecting over 50 million individuals worldwide and representing 60–80% of all dementia cases [5]. Whilst there is evidence that dementia incidence may be declining in the population, potentially attributable to better management of modifiable risk factors, it is not yet known whether this trend will counteract the impact of a shift towards an ageing population [57, 229]. As the population ages, and the risk of developing dementia increases, AD cases are currently set to triple by the year 2050, representing a tremendous global, socio-economic challenge [5]. As existing treatments only target symptoms and do not slow (let alone halt or reverse) the progression of the disease, the need to develop a disease-modifying therapeutic has never been more urgent [155].

Since the identification of microtubule-associated protein tau (MAPT) and amyloid beta (Aβ) as the components of NFTs and extracellular plaques, respectively, research has primarily focused on the toxic roles these proteins play in AD pathogenesis [136, 171, 241]. Accumulation of Aβ causes synapse damage [106, 132, 274] and can induce cognitive and electrophysiological deficits [270]. Similarly, whilst tau was discovered as a microtubule-associated protein (MAP) [276], attention has been drawn to the toxic effects of tau hyperphosphorylation and aggregation [9]. As such, therapeutic development has focused heavily on targeting these proteins through preventing their aggregation, inhibiting their production, or promoting their clearance [155]. Despite promising results in pre-clinical studies, no clinical trials have produced meaningful benefits for patients, with trials being halted due to adverse side effects such as liver toxicity, encephalitis, vasogenic oedema, and even exacerbation of cognitive decline [203, 286]. It is becoming increasingly apparent that the involvement of “pathological” proteins in AD is complex and nuanced [241]. There is mounting evidence that these proteins may serve a number of crucial physiological functions that could be disrupted in the development of AD pathology or by Aβ- or tau-lowering therapeutics.

In this review, we discuss the emerging evidence for key physiological roles of tau and Aβ. The toxic roles of these proteins have been extensively reviewed elsewhere [94, 155, 241], and thus, we aim to highlight possible loss of function phenotypes in disease, as well as identify potentially detrimental side effects of tau- (Table 1) or Aβ (Table 2)-lowering therapeutics if not appropriately targeted. This will be especially important when considering treatment of the adult nervous system, where the lack of developmental compensation may reveal phenotypes masked in constitutive knockouts. We propose that balancing the consideration of both physiological and pathological roles of tau and Aβ will be essential for the design of safe and effective AD therapeutics.

**Table 1:** Adverse effects of lowering tau

Role	Experimental paradigm	Adverse effects	References
**Microtubules** Regulation of microtubule dynamics	Tau knockdown	↓ In labile microtubule mass, ↑ in the stable domain	Qiang et al. [213]
	Tau knockdown	↓ Neuronal outgrowth	Liu et al. [150]
	Tau knockdown	Impaired repulsive response of the growth cone	Biswas and Kalil, [22], Li et al. [147]
	Tau knockdown	Disruption to axonal extension	Caceres and Kosik [36]
	Tau knockdown/ knockout	Delayed neuronal maturation	Caceres et al. [37], Dawson et al. [54]
	Tau knockout	↓ Microtubule density in small caliber axons	Harada et al. [96]
	No tau added to microtubules in vitro (compared to tau presence)	↑ EB1 binding to microtubule ends, ↑ catastrophe frequency	Ramirez-Rios et al. [217]
Regulation of axonal transport	4R tau knockdown	↑ Velocity of mitochondrial axonal transport	Beevers et al. [15]
Protection of microtubules from cleavage	Tau knockdown	Katanin-mediated cleavage, loss of microtubules and ↓ axon length	Qiang et al. [214]
	Tau knockdown	↑ Neuronal branching	Yu et al. [287]
**Synaptic Activity** LTP, LTD and memory	Tau knockout	Age-dependent cognitive deficits in contextual fear conditioning, Y-maze, Morris Water Maze and reversal learning tests	Ahmed et al. [3], Lei et al. [146], Ma et al. [164], Regan et al. [218]
	Tau knockout	Severe LTP deficit	Ahmed et al. [3]
	Tau knockout	LTD deficits	Kimura et al. [128], Regan et al. [218]
	Acute tau knockdown using shRNA	↓ Dendritic spine density, loss of synaptic proteins and significant spatial memory impairments (no compensatory MAP upregulation)	Velazquez et al. [266]
Regulation of neuronal hyperexcitability	Tau knockout	Hyperpolarised neuronal membrane potential	Pallas‐Bazarra et al. [197]
	Tau knockout	Impaired basal neurotransmission when crossed with APP transgenic mouse	Puzzo et al. [209]
Neurogenesis and synaptogenesis	Acute tau knockdown using shRNA	↓ In baseline spine numbers, pro-synaptic response to BDNF blocked	Chen et al. [44]
	Acute tau knockdown using shRNA	↓ Apical and basal dendrite density	Velazquez et al. [266]
	Tau knockout	Failed normal migration of new-born granule neurons in the dentate gyrus	Fuster-Matanzo et al. [78], Sapir et al. [227]
	Tau knockout	↓ Dendritic length, disrupted PSD and mossy fiber terminal formation	Pallas‐Bazarra et al. [197]
	Tau knockout	Impaired neurogenesis	Hong et al. [107]
	Tau knockout	Delayed neuronal maturation	Dawson et al. [54]
	Tau knockout	Transcriptional repression of neuronal genes	de Barreda et al. [11]
**Behaviour** Hyperactivity	Tau knockout	Hyperactivity	Biundo et al. [23], Ikegami et al. [114]
Anxiety	Tau knockout	↑ Rearing behaviour	Lei et al. [146]
	Tau knockout	↑ Anxiety in open field arenas	Gonçalves et al. [86]
Sleep	Tau knockout	↑ Wakefulness and disruption to normal circadian activities	Arnes et al. [8], Cantero et al. [41]
**Motor function**	Tau knockout	FTD-P17-like motor dysfunction	Lei et al. [145]
	Tau knockout	Changes in gait, ↓ locomotion and muscle weakness	Lei et al. [145, 146], Ikegami et al. [114]
	Tau knockout	Loss of dopaminergic neurons	Lei et al. [145], Ma et al. [164]
	Tau knockout, tau 4R knockout, acute tau knockdown using shRNA	Significant impairment in balance beam or rotarod performance	Lei et al. [145, 146], Morris et al. [186], Lopes et al. [157],Ikegami et al. [114],Ma et al. [164],Gumucio et al. [93], Velazquez et al. [266]
**Myelination** Regulation of myelination	Tau knockdown using siRNA	↓ Oligodendrocyte process outgrowth, ↓ myelin basic protein expression, ↓contact with axons	Seiberlich et al. [230]
	Tau knockdown using siRNA	↓ Recovery after sciatic nerve damage, defective myelin debris clearance, impaired Schwann cell migration and differentiation	Yi et al. [285]
	Tau knockout	Age-dependent degeneration of myelinated fibers, ↓ nerve conduction and progressive hypomyelination, resulting in motor and nociceptive impairments	Lopes et al. [157]**,** Sotiropoulos et al. [237]
	Tau knockout	Worse clinical outcome after experimental autoimmune encephalomyelitis (EAE)	Weinger et al. [277]
	Expression of an inducible, truncated tau	Demyelination and development of gait abnormalities	LoPresti [160]
**Response to injury** Promotion of recovery	Tau knockout	↓ Recovery after sciatic nerve damage	Yi et al. [285][]
	Tau knockout	Worse outcome after EAE	Weinger et al. [277]
**Mitochondrial activity** Mitochondrial mobility and health	Tau knockdown	↓ Mitochondrial mobility and ↑ number of abnormal mitochondria	Sapir et al. [227]
**Iron** Regulation of iron homeostasis	Tau knockout	Age-dependent iron accumulation associated with neurodegeneration, cognitive deficits and parkinsonian-like motor deficits, deficits rescued by treatment with the iron chelator clioquinol	Lei et al. [144, 145]
	Lithium-mediated tau reduction	↑ Iron accumulation in the brain, ↓ cellular efflux of iron	Lei et al. [143]
**Nuclear activity** Protection of DNA from damage	Tau knockout	Extensive heat shock damage (DNA breaks) in neurons	Sultan et al. [246]
	Tau knockout	↑ DNA fragmentation under physiological conditions and high susceptibility to DNA breakage after hyperthermic stress	Violet et al. [267]
	Tau knockout	Delayed repair of double-strand breaks after heat shock	Violet et al. [267]
Maintenance of chromosomal stability	Knockout of one or both copies of tau	Marked ↑ in aneuploidy	Granic et al. [88], Rossi et al. [222]
	Tau knockout	Disrupted pericentromeric heterochromatin	Maina et al. [165], Mansuroglu et al. [169]
Regulation of transcription	Tau knockdown using shRNA	↓ mRNA and protein levels of VGLUT1	Siano et al. [234]
	Tau knockout	Upregulation of proteins such as BAF-57 (involved in neuron-specific gene repression)	de Barreda et al. [11]
	Tau knockdown	rDNA transcription altered	Maina et al. [165], Samra et al. [226]
**Tumour suppression**	Tau knockdown	Enhanced cell growth and invasion in clear cell renal cell carcinoma	Han et al. [95]
**Glucose metabolism**	Tau knockout	Insulin resistance in the hippocampus	Marciniak et al. [170]
	Tau knockout	Pancreatic β cell dysfunction and glucose intolerance	Wijesekara et al. [279]

Summary of studies reporting adverse outcomes after lowering tau in a range of experimental systems

**Table 2:** Adverse effects of lowering Aβ

Role	Experimental paradigm	Adverse effects	References
**Synaptic activity** *LTP*	*APP* or *BACE1* knockout	Cognitive deficits induced and impaired LTP	Dawson et al. [53], Laird et al. [139], Lombardo et al. [154], Wang et al. [272, 273]
	Treatment with anti-Aβ antibody 4G8	LTP formation prevented	Morley et al. [185], Puzzo et al. [210]
	Infusion of anti-Aβ antibody 4G8 or siRNA to APP	Short-term memory abolished in contextual fear conditioning or the Morris Water Maze	Garcia-Osta and Alberini [81], Morley et al. [185], Puzzo et al. [210]
	BACE1 inhibitor treatment (wild-type mice)	Suppression of LTP, impaired cognitive performance	Filser et al. [74]
Regulation of neuronal hyperexcitability	*APP* or *BACE1* knockout	Hypersensitivity to spontaneous and induced seizures	Hitt et al. [103], Hu et al. [112], Kobayashi et al. [130], Steinbach et al. [243]
Neurogenesis and synaptogenesis	*APP* knockout	↓ Neuronal branching and synapse formation	Southam et al. [239]
	*APP* knockout	Loss of synaptic proteins	Dawson et al. [53], Seabrook et al. [228]
	*BACE1* knockout	Hearing impairment linked to aberrant synaptic organisation in the cochlea	Dierich et al. [60]
	BACE1 inhibitor treatment (wild-type mice)	↓ Spine density, ↓ spine formation	Filser et al. [74]
**Myelination** Regulation of myelination	*BACE1* knockout	Delayed myelination, ↓ myelin thickness	Hu et al. [110], Willem et al. [280]
	*BACE1* knockout	Impaired remyelination of peripheral nerves after injury	Hu et al. [109, 111]
**Role in blood vessels** Promotion of angiogenesis	*BACE1* knockout	↓ In retinal vascular density	Cai et al. [38]
	APP-deficiency or BACE1 inhibitor treatment	Shorter hindbrain vessels, fewer cerebrovascular branches	Luna et al. [163]
	γ-secretase inhibitor treatment	↑ Angiogenesis and vascularisation	Cameron et al. [40]
“Vascular plug”	Aβ-targeting drugs (active or passive Aβ immunisation) in human clinical trials	Microhaemorrhages and brain oedema (“Amyloid-Related Imaging Abnormalities” (ARIA))	Penninkilampi et al. [203], Sperling et al. [240]
	Aβ immunisation (animal models)	ARIA-like cerebral microbleeds	Blockx et al. [24], Joseph-Mathurin et al. [121]
	*APP* or *BACE1* knockout	↑ Mortality after ischaemic injury, deficits in reactive blood flow	Koike et al. [133]
**Response to injury** Promotion of recovery	*BACE1* knockout	Impaired remyelination after sciatic nerve lesion	Hu et al. [109, 111]
	*BACE1* knockout	Worse functional outcome after spinal cord injury	Pajoohesh-Ganji et al. [195]
	*BACE1* knockout	Worse outcome after controlled cortical impact (rescued by Aβ application)	Mannix et al. [167, 168]
	*BACE1* or *APP* knockout	↑ Risk of mortality following cerebral ischaemia	Koike et al. [133]
**Antimicrobial activity**	*APP* knockout	↑ Mortality after infection	Kumar et al. [138]
	Aβ-targeting therapies	↑ Incidence of infections	Gosztyla et al. [87]
**Iron homeostasis** Regulation of iron homeostasis	*APP* knockout	↑ Neuronal iron retention in vitro, ↑ vulnerability to oxidative damage from dietary iron in vivo	Duce et al. [63]
	*APP* knockout	Age-dependent iron accumulation in the brain and liver	Belaidi et al. [16]
**Glucose metabolism**	*BACE1* knockout	↓ Insulin expression in the pancreas	Hoffmeister et al. [104]
	BACE1 knockdown (siRNA)	↓ Insulin mRNA and protein in insulinoma cells	Hoffmeister et al. [104]

Summary of studies reporting adverse outcomes after lowering Aβ in a range of experimental systems

## The origin of Aβ and tau

### Aβ biogenesis

**Fig. 1:**
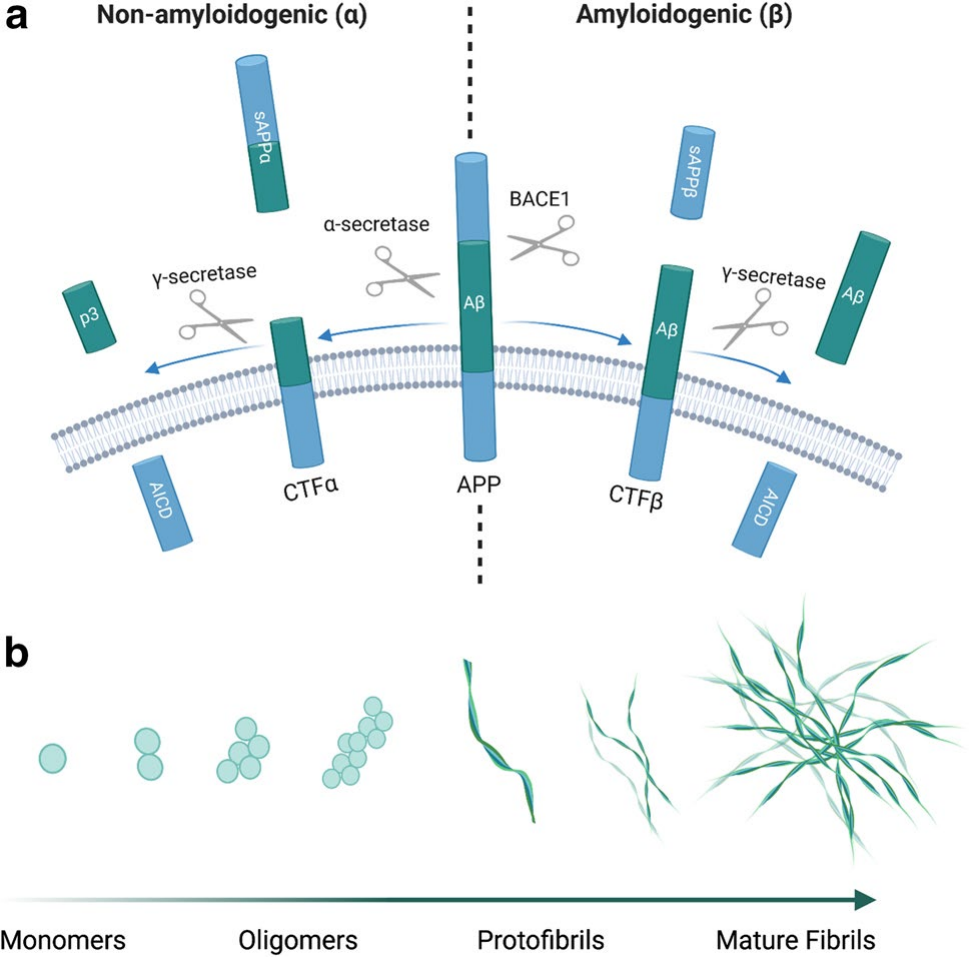
**a** The two pathways through which APP can be cleaved. The non-amyloidogenic (α) pathway (left-hand side of diagram) involves the cleavage of APP by α-secretase, within the Aβ sequence, to form C-terminal fragment α (CTFα) and soluble APP α (sAPPα). γ-secretase then cleaves the resulting CTFα, releasing the Aβ intracellular domain (AICD) and the extracellular p3 fragment. The amyloidogenic (β) pathway (right-hand side of the diagram) involves the cleavage of APP by BACE1 to form CTFβ and sAPPβ. γ-secretase then cleaves the resulting CTFβ, releasing the AICD and Aβ. **b** Aβ monomers can assemble to form higher order structures, from oligomers, to protofibrils and eventually mature fibrils containing β-sheets which form the core component of amyloid plaques. Created with https://biorender.com/

Amyloid precursor protein (APP) is encoded by the *APP* gene on chromosome 21 [84, 125, 257]. Aβ is produced by the sequential cleavage of APP by beta-site amyloid precursor protein cleaving enzyme 1 (BACE1) [265] and γ-secretase [55], which have recently been shown to form a multiprotease complex to maximise cleavage efficiency [151] (Fig. 1a). BACE1 cleaves APP at Asp1 or Glu11 of the Aβ sequence [265], after which the catalytic component of γ-secretase (presenilin) sequentially trims the resulting carboxy-terminal fragment at ε-, ζ-, and γ-cleavage sites, releasing the Aβ intracellular domain (AICD) and Aβ (between 37 and 49 amino acids in length) [292]. Mutations in presenilin, or environmental factors, consistently decrease the processivity of γ-secretase, resulting in fewer cuts per APP molecule and consequentially the release of longer Aβ peptides [42]. This amyloidogenic (or β) cleavage of APP is in direct competition with an alternative α-cleavage pathway, where α-secretase bisects APP in the middle of the putative Aβ protein, thus preventing Aβ production [70]. Under physiological conditions, Aβ_1-40_ (~ 90% of total Aβ) and Aβ_1-42_ (~ 5–10% of total Aβ) are the most abundant isoforms in humans [184]. Aβ_1-40_ is produced exclusively within the trans-Golgi network (TGN) and then packaged into secretory vesicles, whilst Aβ_1-42_ can be made in either the TGN or the endoplasmic reticulum [91]. Aβ monomers readily assemble to form higher order structures, from low-molecular-weight oligomers, to protofibrils and eventually to fibrils containing β-sheets (Fig. 1b), with longer isoforms of Aβ showing the greatest propensity to oligomerise and aggregate [34]. Increased production of Aβ_1-42_, at the expense of Aβ_1-40_ generation, is a common feature of both familial [42] and sporadic AD [101], with the increased aggregation of this peptide believed to be responsible for driving neurotoxicity [34].

### Tau production

**Fig. 2:**
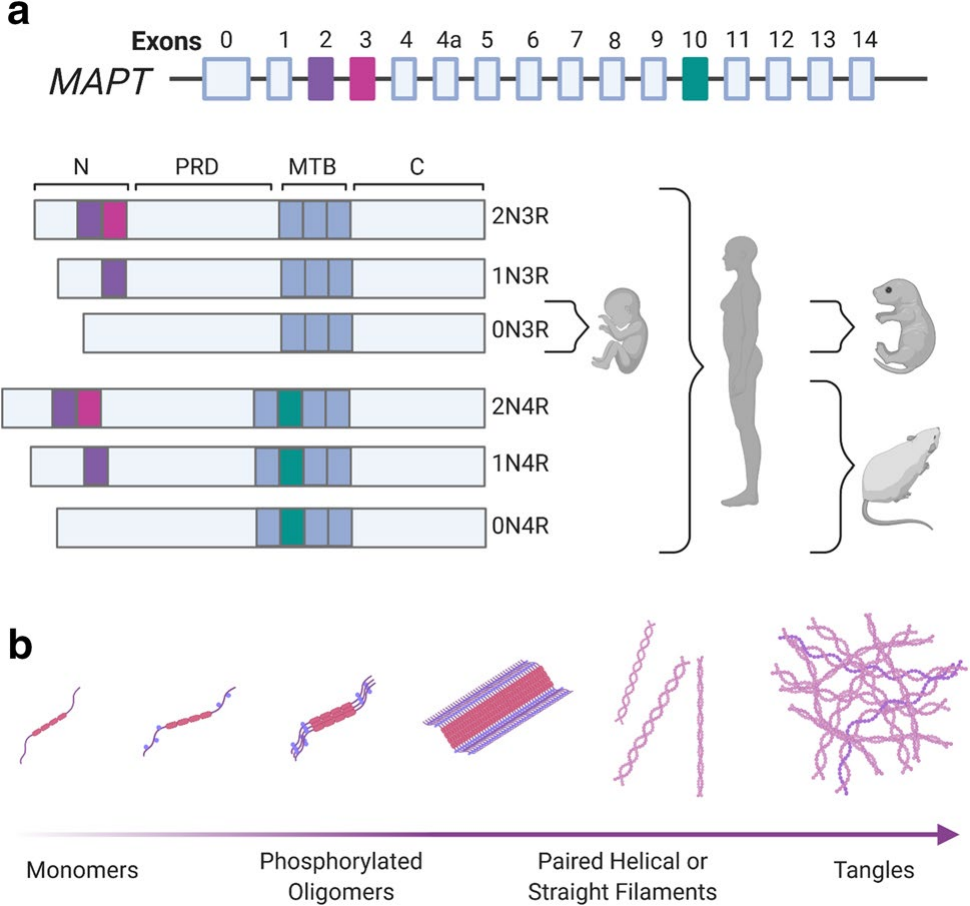
**a** Tau is encoded by the *MAPT* gene on chromosome 17. A total of 6 tau protein isoforms are generated via alternative splicing of exons 2, 3, and 10. Inclusion of exon 10 produces tau with 4 microtubule-binding (MTB) domains (4R), with omission of exon 10 producing tau with 3 MTB domains (3R). Tau can include (2 N or 1 N) or exclude (0 N) amino-terminal inserts through regulation of exons 2 and 3. Only 0N3R tau is expressed in the foetal human or mouse brain, with all 6 tau isoforms being expressed in adult humans. Adult mice and rats show almost exclusive expression of 4R tau. **b** Phosphorylated tau monomers can assemble to form oligomers, filaments (both straight and paired helical) and eventually tangles. ***N*** N-terminus, *PRD* proline-rich domain, *MTB* microtubule-binding domains, ***C*** C-terminus. Created with https://biorender.com/

Tau is encoded by the *MAPT* (microtubule-associated protein tau) gene on chromosome 17 [189], which generates a total of 6 tau protein isoforms through alternative splicing of exons 2, 3, and 10 in the central nervous system (CNS) [94] (Fig. 2a). Inclusion of exon 10 produces tau with 4 microtubule-binding domains (4R), whilst omission of exon 10 excludes microtubule-binding domain R2 (3R). Similarly, tau can include (2 N or 1 N) or exclude (0 N) amino-terminal inserts through regulation of exons 2 and 3. In the peripheral nervous system (PNS), exons 4A, 6, and 8 can also be transcribed, resulting in the production of larger tau proteins [75]. Tau expression is developmentally regulated, with only 0N3R tau being expressed in the foetal brain, whilst all isoforms are expressed in adult humans [94]. However, adult mice and rats show almost exclusive expression of 4R tau [94]. In humans, there are two principle genetic haplotypes at the *MAPT* locus; H1, which is directly orientated (~ 75% of the Caucasian population), and H2, which has an inverted sequence (~ 25% of the Caucasian population) [269]. Interestingly, the H2 haplotype appears to be almost exclusively Caucasian in origin, with Central Asian populations having H2 allele frequencies of ~ 5% and African, East Asian, and Native American populations effectively lacking H2 expression [71]. Possession of the H1 versus H2 haplotype subtly alters the tau isoform expression profile [269]. Tau can undergo a vast array of post-translational modifications including phosphorylation, acetylation, ubiquitination, sumoylation, methylation, glycation, glycosylation, polyamination, nitration, isomerisation, and oxidation (reviewed in [94]). Tau monomers can aggregate to form oligomers and higher order fibrils (Fig. 2b). However, whilst Aβ can largely self-assemble, tau phosphorylation is believed to be important for its aggregation [9].

### Aβ and tau are expressed throughout the body

**Fig. 3:**
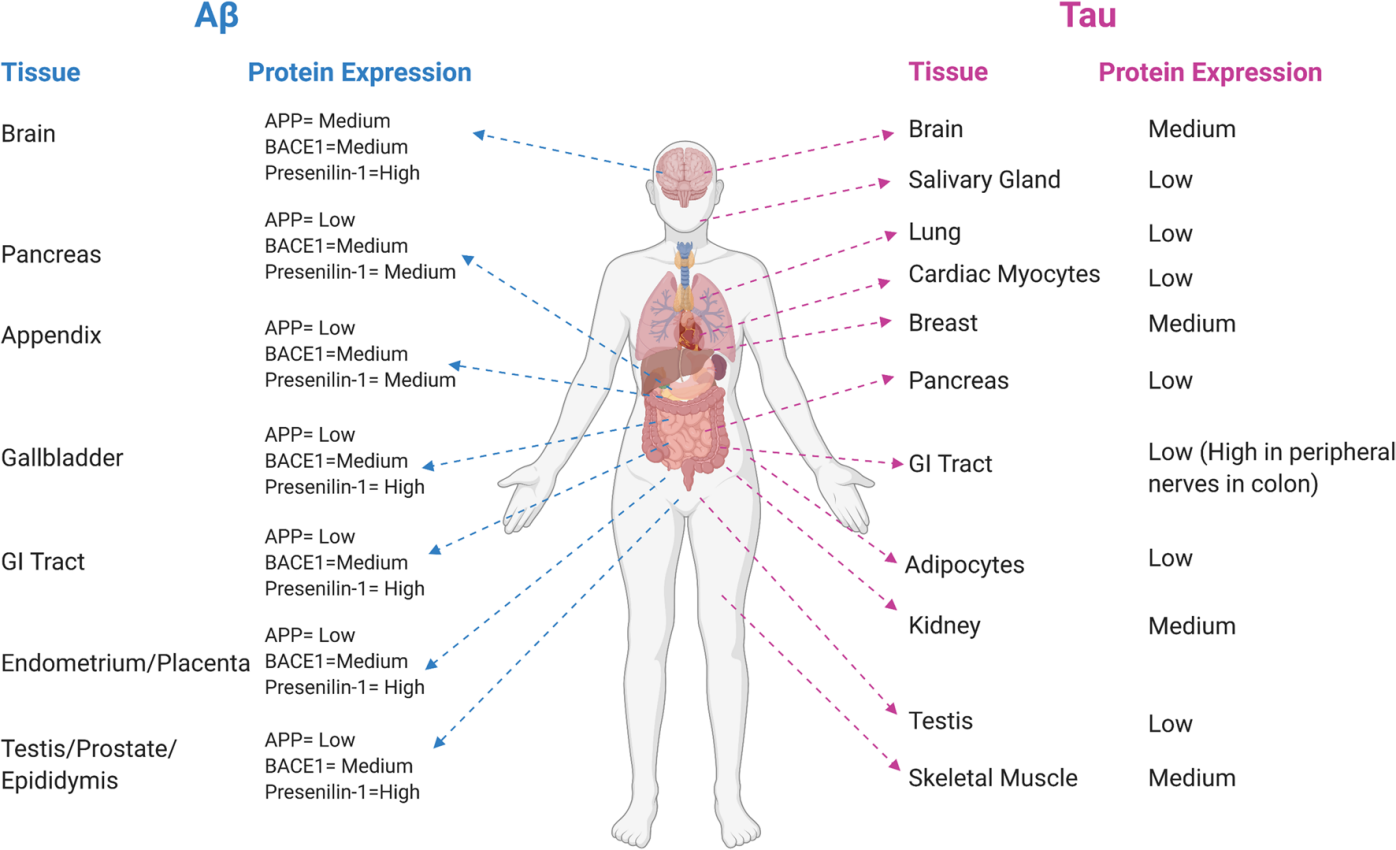
Tissue-level protein expression of APP [297], BACE1 [298], and presenilin-1 [299] (indicating potential for Aβ production) and tau (MAPT) [296] according to the Human Protein Atlas [262]. Created with https://biorender.com/

Tau and the key proteins required to produce Aβ [APP, BACE1, and components of γ-secretase (presenilin-1)] are expressed in a variety of tissues throughout the body (as reported by The Human Protein Atlas [262] (Fig. 3)). Whilst tau is predominantly found in brain and peripheral nerves, tau protein expression has been detected in diverse locations including salivary glands, breast tissue, cardiac myocytes, skeletal muscle, the pancreas, and kidneys [296]. Similarly, in addition to the brain, the key components of the Aβ-processing pathway [APP, BACE1, and presenilin-1 (PS1)] are co-expressed in the pancreas, appendix, gastrointestinal tract, and both male and female reproductive organs [297–299]. It is likely, therefore, that both Aβ and tau will serve functions beyond the CNS.

### Aβ and tau are evolutionarily conserved

Both tau and Aβ show remarkable evolutionary conservation. An Aβ-like sequence has been reported in sea anemones, demonstrating that such peptides have been in existence from 540 to 630 million years ago [259]. The human Aβ sequence is over 95% homologous to that in other mammals, and over 90% in birds, reptiles, and amphibians [259]. Similarly, evidence of a *MAPT*-like gene has been found in lampreys, hagfish, and sharks, pinpointing an origin over 550 million years ago [247]. Such evolutionary conservation raises the possibility that these proteins are involved in key biological functions. Identifying these functions will provide a greater understanding of the pathogenesis of AD, as well as informing how Aβ- or tau-targeting treatments could impact physiology.

## Regulation of microtubules: the primary physiological role of tau?

### Tau binds to and regulates the structure of microtubules

Since the discovery of tau in 1975 [276], a plethora of research has focused on the role of tau at microtubules (reviewed in [10]). Microtubule deficits are common in AD and related disorders, with studies reporting axonal transport deficits [1], and defective microtubule assembly [115]. Tau binds tubulin via its microtubule-binding domains [141], with a single tau molecule crosslinking multiple tubulin dimers [6]. Original studies found that tau stabilises microtubules [62], reducing the frequency of catastrophes (sudden microtubule disintegration) [208]. Early studies found that tau reduces the concentration of tubulin required for polymerisation [276]. A mechanism for this process has recently been proposed, where conditions of macro-molecular crowding induce tau to form liquid-like drops [102]. Tubulin partitions into these drops, effectively raising its concentration to drive nucleation of microtubule formation [102]. Crucially, tau from AD post-mortem brain tissue fails to stimulate microtubule formation [115] and hyperphosphorylation of tau [115, 192], mutations within microtubule-binding sites [83], or C-terminal truncation [192], all greatly reduce tau’s microtubule-binding capacity. These findings have contributed to the established dogma that disease-associated hyperphosphorylation of tau promotes its dissociation from microtubules, reducing microtubule stability [9]. Whilst pseudo-phosphorylation of tau results in a fivefold decrease in the association rate of binding to microtubules, this surprisingly does not impact tau’s *dissociation* rate, raising the question of whether tau hyperphosphorylation occurs prior to, or following, microtubule detachment in disease [192]. In support of the latter hypothesis, inducing microtubule catastrophe via stathmin application results in unphosphorylated tau falling off the microtubule and subsequently being phosphorylated in the cytoplasm [181]. Thus, the role of microtubule-associated tau appears to be more complex than originally thought, with recent studies demonstrating its diverse, subtle, and sometimes contradictory functions.

### Beyond microtubule stability: tau as a regulator of microtubule dynamics

Tau is preferentially expressed within the axon and there is agreement that tau concentration increases towards the distal, labile end [213]. As such, a role for regulating microtubule *dynamics* has been proposed. Indeed, tau knockdown in primary neurons results in a substantial drop in the labile microtubule mass with a corresponding increase in the stable domain [213]. Tau knockdown reduces neuronal outgrowth [150], impairs the repulsive response of the growth cone [22, 147], disrupts axonal extension [36], delays neuronal maturation [37, 54], and reduces microtubule density [96]. Tau also recruits end-binding proteins (EBs) to the stable microtubule bundle, preventing them from tracking to microtubule ends where they increase catastrophe frequency [217]. However, not all tau depletion studies report deficits in microtubule dynamics [261], with compensatory increases in other MAPs potentially masking relevant functions [164, 266]. Indeed, *MAPT* and *MAP1B* double knockouts have a high mortality rate, showing a synergistic disruption to growth cone dynamics, axonal elongation, and neuronal migration, resulting in defective axonal tract and neuronal layer formation [252]. Interestingly, an individual with frontotemporal dementia (FTD) was found to have a partial deletion of the *MAPT* gene, resulting in the production of truncated tau lacking the first microtubule-binding repeat [224]. This truncated tau exhibited dramatic reduction in microtubule-binding capability, but acquired the ability to sequester MAP1B, potentially mediating both loss-of-function and gain-of-function disruption to microtubule dynamics, similar to double knockout mice [224].

### Tau regulates axonal transport

Many studies have sought to determine whether tau regulates axonal transport. Knockdown of 4R tau in human induced pluripotent stem cell (iPSC)-derived neurons *increases* the velocity of mitochondrial transport [15]. Additionally, young P301L knockin mice, which exhibit reduced tau-microtubule binding, show enhanced anterograde transport [1, 83]. It is hypothesised that tau may compete with motor proteins for tubulin-binding sites, and overexpression of tau has been reported to cause “traffic jams” [242] and induce kinesin dissociation from microtubules [61, 65, 233, 255]. Tau fibrils or oligomers can also inhibit axonal transport [124] and *MAPT*^*−/−*^ neurons are resistant to Aβ-induced axonal transport deficits [268]. Recent work has demonstrated a potential mechanism for this under physiological conditions, with two studies describing “island” regions of concentrated tau protein along the axon [233, 255]. Upon reaching the island boundaries, kinesin-1 motor proteins dissociate instantaneously from the microtubules, whilst dynein slowly moves through [233, 255]. Such differential regulation of motor proteins could allow tau to regulate axonal transport and cargo delivery, processes that may be disrupted in disease. However, other studies report that neither tau knockout nor overexpression alters axonal transport dynamics [288]. A “kiss-and-hop” binding of tau to tubulin has been proposed as a mechanism, whereby tau remains associated with microtubules without clogging motor protein binding sites [118].

### Tau protects microtubules from cleavage

An emerging role of tau is protecting microtubules from the microtubule-severing protein katanin [214, 233, 255, 287]. Recently described “tau islands” block katanin-tubulin binding, preventing breakdown of the microtubule lattice at these sites [233, 255]. Indeed, tau knockdown [214], or expression of pseudo-hyperphosphorylated tau [245], results in katanin-mediated cleavage, loss of microtubules, and reduced axon length in vitro. Tau depletion also increases neuronal branching [287], raising the hypothesis that the preferential expression of tau in the axon may maintain a non-branched structure, in contrast to highly branched dendrites, which show low tau expression [287]. Pathological tau mislocalisation may, therefore, render axonal microtubules vulnerable to inappropriate cleavage [233]. Interestingly, Aβ-induced tau mis-sorting permits recruitment of tubulin tyrosine ligase-like 6 (TTLL6) to dendritic microtubules, promoting spastin-mediated cleavage [289]. Tau *location*, therefore, appears vital for regulating its physiological versus pathological functions. Indeed, dendritic mis-sorting of tau also promotes aberrant clustering of Fyn, a key step in Aβ-mediated synaptotoxicity and spine collapse [116].

### The future of therapeutics targeting microtubule (dys)function

Whilst microtubule-stabilising drugs, such as Taxol and Epothilone D, showed pre-clinical promise, replication of this success in humans is not yet forthcoming [286]. Forty five years of exploration of tau at the microtubule has transformed understanding from tau being a simple “stabiliser”, to reveal a diverse array of physiological and pathological functions [10]. With development of tau-targeting therapies becoming increasingly common, careful maintenance of physiological tau at the axon, whilst also preventing mis-sorting and pathological aggregation, will likely be crucial for therapeutic success. Interestingly, a recent study reported that Aβ-mediated dendritic simplification requires microtubule stabilisation by unphosphorylated dendritic tau [85]. This finding raises concerns that reducing tau phosphorylation, a common goal of tau-targeting treatments, may negatively impact neuronal connectivity in some circumstances, highlighting the complexity of untangling physiological from pathological modifications. Therefore, focus on other physiological functions of tau or Aβ may reveal more promising therapeutic targets.

## Physiological roles at the synapse

Synapse loss is the strongest pathological correlate of cognitive decline in AD [56, 258], so protecting these vital structures is a key therapeutic goal. Tau and Aβ are prime suspects for causing synaptic damage, with toxic species reported to accumulate at synapses [132, 250], disrupt key synaptic machinery [293], induce spine collapse [274], and target synapses for microglia-mediated pruning [52, 106]. Whilst toxic tau and Aβ are undoubtedly involved in AD pathology, there is growing evidence that these proteins play key *physiological* roles at the synapse that could be lost, or hijacked, to contribute to disease. Potential loss-of-function at the synapse should be carefully considered when designing tau- or Aβ-targeting therapeutics to avoid exacerbating, or introducing new, synaptic pathology.

### Physiological concentrations of Aβ enhance LTP

**Fig. 4:**
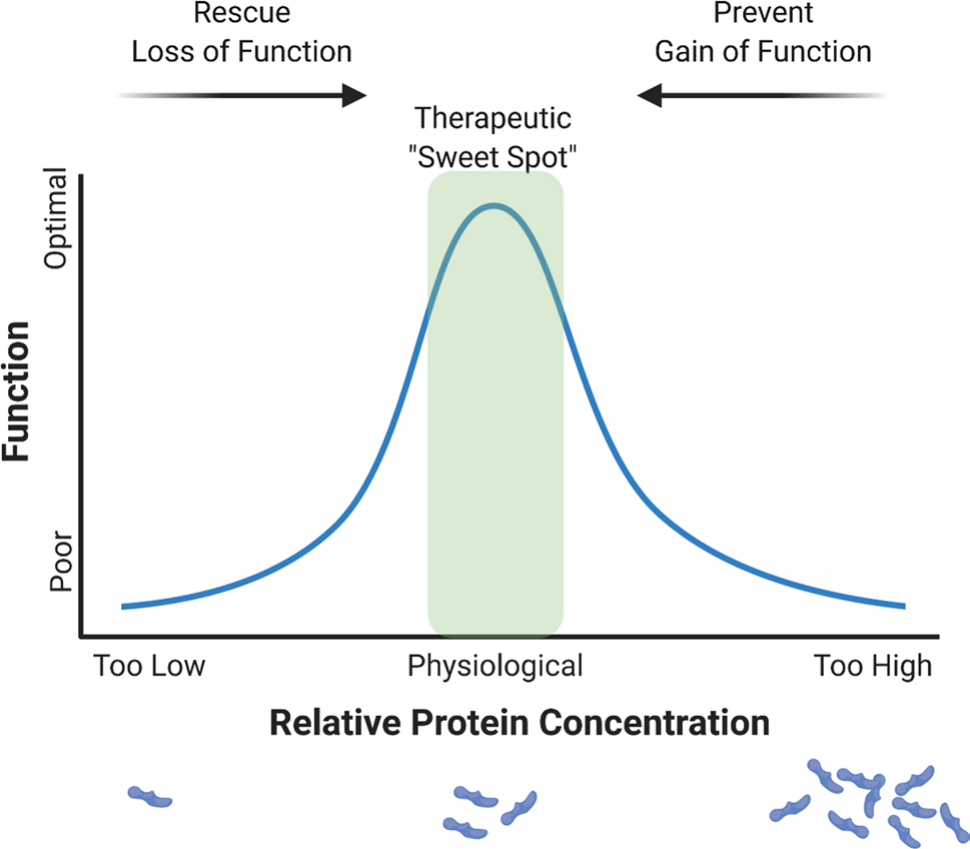
Schematic representation of the hormetic responses to tau and Aß concentration. There is an optimal concentration of tau or Aβ for a number of physiological functions. Too little protein (or loss of function modifications) or too much protein (or gain of function modifications) can both disrupt normal function. Effectively rescuing loss of function or preventing gain of function to maintain optimal physiological conditions should be the ultimate goal of therapeutics. Created with https://biorender.com/

Studies of synapse function often use long-term potentiation (LTP), an indicator of synapse strengthening, and long-term depression (LTD), an indicator of synaptic weakening, as electrophysiological correlates of learning and memory [14]. Pathological concentrations of Aβ applied to hippocampal slices disrupt LTP [270], but physiological concentrations may, in fact, be required for this process [210] (Fig. 4). A biphasic, or hormetic, role of soluble Aβ in regulating LTP has been proposed, whereby low (picomolar) concentrations of Aβ *enhance* LTP, whilst high (nanomolar) concentrations suppress LTP [92, 99, 185, 210, 211]. Such experiments highlight a potential role for endogenous Aβ in regulating memory formation. Indeed, the extracellular concentration of Aβ increases after neuronal stimulation [123, 253] and interstitial fluid Aβ concentration positively correlates with neuronal activity in human brain [29]. Interestingly, both *APP* knockout [53] and *BACE1* knockout [139, 154, 272, 273] in vivo induce cognitive deficits and impair LTP. Wild-type mice given BACE1 inhibitors also show a dose-dependent suppression of LTP and impaired cognitive performance [74]. Treatment of wild-type slices with the anti-Aβ antibody 4G8 prevents LTP formation [185, 210] with LTP restored by application of picomolar oligomeric Aβ_1-42_ [210]. In vivo*,* hippocampal infusion of 4G8 [81, 185, 210] or siRNA to APP [185, 210] abolishes short-term memory in contextual fear conditioning [81, 185, 210] or the Morris Water Maze (MWM) [210]. Infusion of picomolar Aβ rescues this effect [81, 210], whilst Aβ-infusion into naive mice *enhances* reference and contextual fear memory [81, 210, 211]. A potential hormetic role of Aβ raises the possibility that physiological, learning-mediated Aβ production throughout life could eventually result in accumulation of Aβ to a toxic level, especially if clearance mechanisms fail. Indeed, in humans, brain regions in the default mode network that show high levels of neuronal activity in young adults closely correlate with regions most heavily burdened by amyloid in AD later in life [32].

### Tau in LTP, LTD, and memory

Like Aβ, tau expression and secretion also increases following neuronal activity [131, 207], and studies in *MAPT*^*−/−*^ mice have sought to explore the role of tau in synaptic function. Phenotypes of *MAPT*^*−/−*^ mice vary, with findings differing depending on the model used, genetic background [146], cleanliness of the animal unit [186], diet [164], and the age of the animals when studied. Some studies do not report cognitive or synaptic deficits in *MAPT*^*−/−*^ mice [113, 146, 186, 254], and reports that there is compensatory upregulation of other MAPs highlight that developmental compensation could mask relevant effects [164]. Ma et al. found that 8-month old *MAPT*^*−/−*^ mice had no cognitive deficits, but displayed significant MAP1A upregulation. At 19 months old, however, compensatory MAP levels fell, and extensive synapse loss and cognitive deficits became apparent [164]. A recent study showed that *acute* tau knockdown in adult mice using viral shRNA resulted in reduced spine density, loss of synaptic proteins, and significant spatial memory impairments in the absence of compensatory MAP upregulation [266]. Despite potential compensatory confounds, different groups have reported age-dependent cognitive deficits in *MAPT*^*−/−*^ mice in contextual fear conditioning [3], Y-maze [146], MWM [164], and reversal learning tests [218]. Whilst one study found a severe LTP deficit in tau knockouts [3], LTD deficits are more commonly reported [128, 218]. Tau’s role in LTD appears to depend on its phosphorylation at serine 396 permitting AMPA receptor internalisation [218]. Crucially, a number of studies have reported human genetic cases of FTD [199, 294] or intellectual disability [172, 232, 263] in which tau levels are drastically *reduced* compared to age-matched controls. Reduced soluble tau has also been reported in normal ageing [187], Parkinson’s disease (PD) [145], and AD [137, 191] (potentially due to sequestration of tau into NFTs). Together, this raises the possibility that loss of normal tau function may be partly responsible for cognitive and synaptic deficits in disease. Tau-targeting therapeutics should, therefore, be carefully managed to avoid disrupting physiological tau at the synapse.

### Regulating neuronal hyperexcitability: could tau and Aβ act at opposite ends of the spectrum?

A common feature of many neurodegenerative and neurodevelopmental disorders is the prevalence of seizures. Up to 22% of individuals with AD experience at least one seizure [178], whilst epilepsy is diagnosed in around 20% of individuals with autism spectrum disorder (ASD) [18]. Tau appears to be important for permitting seizure activity, and there is strong evidence that *MAPT*^*−/−*^ mice [148], or mice treated with tau antisense oligonucleotides [59] are resistant to pentylenetetrazol-induced seizures. Crossing *MAPT*^*−/−*^ mice with genetic models of epilepsy [105], AD [219] and ASD [249] can rescue hyperexcitability and spontaneous epileptiform activity. As such, a role for tau in promoting or regulating neuronal excitability seems likely. It has recently been shown that hyperexcitation increases tau translation [131] and that tau translocation to the nucleus regulates the expression of the glutamatergic transporter protein, VGLUT1 [234]. Interestingly, *MAPT*^*−/−*^ neurons lack extrasynaptic NMDA currents that, whilst their physiological role is under debate, mediate excitotoxicity under a number of conditions [196]. Tau deficiency may also impact basal synaptic activity; one study reported that tau knockout neurons were hyperpolarised compared to wild-type cells [197], and a recent study found that tau knockout impaired basal neurotransmission in APP transgenic mice [209]. Lowering of tau levels could, therefore, be beneficial under conditions of hyperexcitability, but this must be carefully balanced against the risk of depressing normal neuronal activity.

When considering the impact of Aβ on neuronal excitability, there are seemingly contradictory findings, potentially explainable through the aforementioned hormetic role of Aβ (Fig. 4). Whilst low levels of Aβ can increase dendritic spine density [194], increase the number of docked vesicles [92], enhance glutamate release, promote excitotoxicity, and disrupt calcium homeostasis (particularly in early stages of disease) [7, 35, 225], Aβ production after synaptic activity could also act via negative feedback to prevent hyperactivity [123]. Indeed, Aβ can induce spine collapse and synapse loss [231, 274], increase the proportion of silent neurons [35], and disrupt neurotransmitter release via depletion of presynaptic PIP_2_ [99]_._ However, untangling the specific role of Aβ versus APP, alternative APP-processing products or BACE1 has proven difficult. Both *APP*^−/−^ [243] and *BACE1*^−/−^ [103, 112, 130] mice fail to produce Aβ and show hypersensitivity to spontaneous and induced seizures. BACE1, however, cleaves a variety of proteins important for normal neuronal function, including seizure protein 6 [206]. Notably, conditional *BACE1* knockout in adult mice does not induce epileptiform activity [264], suggesting BACE1 or Aβ may play independent roles in the developing versus adult brain. To add further complexity, overexpression of APP, not Aβ, is responsible for hypersynchronous activity in some AD mouse models [26]. Therefore, clarification of the roles of Aβ, APP, and BACE1 will be essential to ensure balanced synaptic activity when therapeutically targeting amyloid dysregulation.

### Neurogenesis, synaptogenesis, and structural plasticity

The formation of new neurons and synapses is an important process both throughout development and in the adult nervous system. Early studies found that low concentrations of Aβ promote the survival of primary neurons [278] and have a neurogenic effect on neural progenitor cells [158]. Aβ_1-42_ also stimulates neurogenesis of subventricular zone precursors in vivo, raising the hypothesis that the early overstimulation of neurogenesis in AD may result in depletion of the stem cell pool and a decline in basal neurogenesis later in life [238]. Despite showing neurogenic properties, Aβ is often considered an antagonist to the formation of new synapses. Indeed, high concentrations of oligomeric Aβ can induce spine collapse [7, 231, 274], and synapse loss has been found to correlate with increased intraneuronal APP [295], plaque-proximal extracellular Aβ [132, 295], and accumulation of intraneuronal Aβ [97, 253]. However, long exposure to picomolar Aβ has also been found to increase the spine density in slice cultures [194]. Similarly, *APP* knockout in hippocampal neurons reduces neuronal branching and synapse formation [239], and *APP*^*−/−*^ mice show a profound loss of synaptic proteins [53, 228], although loss of sAPPα (a non-amyloidogenic product of APP cleavage) may be a key contributor to this phenotype (reviewed in [47]). BACE1 has also been shown to play a role in normal synapse development, with *BACE1*^*−/−*^ mice showing a developmental hearing impairment caused by aberrant synaptic organisation in the cochlea [60]. Interestingly, adult wild-type mice treated with BACE1 inhibitors also show a reduction in dendritic spine density and formation [74], whilst BACE1 inhibitor treatment of APP/PS1 mice (which produce excess Aβ) slowed the rate of synapse loss around plaques [204]. Taken together, it once again seems likely that there is an optimal concentration of Aβ to maintain synapses, which must be considered when therapeutically targeting Aβ (Fig. 4).

Tau may also play a role in neuronal development and synaptogenesis. In vitro*,* stimulation of hippocampal neurons with brain-derived neurotrophic factor (BDNF) normally increases tau expression and spine growth, but tau-shRNA treatment significantly decreases baseline spine numbers and blocks the pro-synaptic response to BDNF [44]. In adult mice, tau-shRNA treatment reduced apical and basal dendrite density, supporting a role for tau in spine formation or maintenance [266]. In *MAPT*^*−/−*^ mice, new-born granule neurons in the dentate gyrus fail to migrate normally [78, 227], show reduced dendritic length, and have disrupted postsynaptic density (PSD) and mossy fiber terminal formation [197]. Tau also mediates the pro-neurogenic effect of environmental enrichment on adult hippocampal neurogenesis and synaptic integration [197]. Other studies have reported impaired neurogenesis in *MAPT*^*−/−*^ mice [107], potentially explaining the reduced brain weight reported in some strains [145, 146]. An anti-aggregant tau mouse model shows increased neurogenesis and hippocampal volume [120], whilst tauopathy models frequently show deficits in hippocampal neurogenesis [134], potentially highlighting tau aggregation as a loss-of-function mechanism in this context. Tau knockdown also delays neuronal maturation in primary neurons [54] and induces transcriptional repression of neuronal genes [11], indicative of a potential role of tau in promoting neuronal differentiation. Some studies, however, contradict these findings, reporting increased neurogenesis in tau-deficient animals [50]. Further exploration of the role of tau in adult neurogenesis, and whether this translates to humans, is, therefore, required.

## Regulating behaviour

### Tau, hyperactivity, anxiety, and sleep

In addition to cognitive deficits, *MAPT*^*−/−*^ mice show evidence of hyperactivity, as measured by spontaneous locomotion in an open field test [23, 114], and substantially increased rearing behaviour [148]. *MAPT*^*−/−*^ mice also spend significantly less time in the open arms of elevated Z-mazes and keep to the periphery of open field arenas, indicative of increased anxiety [86]. Tau may also help to regulate circadian behaviours, with *MAPT*^*−/−*^ mice [41] or *Drosophila* [8], showing major abnormalities in the sleep–wake cycle, including increased wakefulness and disruption to normal circadian activities. Sleep disturbances are commonly reported in ageing and neurodegenerative disorders [281], so understanding how tau alterations impact sleep may be clinically relevant. Interestingly, tauopathy models often show disturbed sleep patterns [122] and hyperactivity [122, 205] phenotypes, raising the possibility that tau mutations could, for these behaviours, mimic the effect of tau depletion through loss of function.

## Tau and the motor system

Another common feature of *MAPT*^*−/−*^ mice is the appearance of FTD-P17-like motor dysfunction, suggesting that tau loss of function could be partly responsible for Parkinsonism in these individuals [145]. Notably, soluble tau levels in the substantia nigra of the brains of individuals with PD are 44% lower than those observed in age-matched controls, indicating that loss of normal tau may contribute to motor deficits [145]. In mouse studies, a number of groups have reported significant impairment in balance beam or rotarod performance in tau knockouts [114, 145, 146, 157, 164, 186], mice lacking 4R tau [93], and mice treated with tau-shRNA [266]. Additionally, changes in gait [146], reduced locomotion [145, 146], and muscle weakness [114] have been recorded, and many *MAPT*^*−/−*^ models also show loss of dopaminergic neurons [145, 146, 164]. Interestingly, treatment of *MAPT*^*−/−*^ mice with L-DOPA rescued motor phenotypes in some models [145, 146], but another study reported a dopamine-independent motor deficit [186]. A recent study postulated that the age-dependent motor deficits observed in their *MAPT*^*−/−*^ model were due to hypomyelination and degeneration of the sciatic nerve (discussed below), raising further questions about the effect of tau loss of function in the peripheral versus central nervous systems [157]. Interestingly, motor deficits are common in tau-overexpressing transgenic mice [4, 77], and it has been reported that whilst modest overexpression of FTD-P17 mutant tau can improve motor performance in young animals, this progresses to severe paraparesis in later life [183]. In humans, the *MAPT* H1/H1 genotype is also associated with increased risk of neurodegenerative diseases affecting the motor system, such as corticobasal degeneration (CBD) and progressive supranuclear palsy (PSP) [108]. Taken together, it seems likely that, in addition to mutations potentially disrupting physiological roles of tau, changes in tau concentration may have a hormetic effect on motor function (Fig. 4). Whilst individuals with AD can develop motor symptoms [155], this is not considered the primary deficit, indicative that the location of tau changes within the brain (e.g., hippocampus versus substantia nigra or motor cortex) and peripheral nervous system is likely an important determinant of symptoms.

## Regulation of myelination

### Tau is important for normal myelination

Whilst many studies focus on neuronal damage, white matter hyperintensities (evidence of demyelination) are commonly reported in the early stages of AD and other tauopathies and correlate with tau burden [173]. A recent study describes human familial globular glial tauopathy, linked to *MAPT* mutations, that result in extensive tau deposits in oligodendrocytes [73]. Affected individuals show severe demyelination, defective myelin synthesis, and concomitant axonal damage [73]. Coiled bodies of tau in oligodendrocytes and demyelination are also common features of other primary tauopathies such as PSP and CBD [291]. Mouse models of tauopathy frequently demonstrate deficits in myelination, with evidence of filamentous tau inclusions developing in oligodendrocytes [149], resulting in defective myelination of the perforant path [117], spinal cord [149], and sciatic nerve [179]. Tauopathy-induced myelination deficits coincide with increased nerve conduction latency [117], cognitive decline [117], and motor impairments [179]. The mechanism by which tau pathology leads to deficits in myelination has yet to be fully elucidated, but there is evidence that this could represent a loss of normal tau function. Tau is found within oligodendrocytes and Schwann cells under physiological conditions and is upregulated during developmental myelination [159]. siRNA-mediated knockdown of tau impairs oligodendrocyte process outgrowth, reduces myelin basic protein (MBP) expression, and impairs contact with axons in myelinating co-cultures [230]. Oligodendrocyte process outgrowth depends on the recruitment of tau and tubulin to activated Fyn-kinase rafts, with disruption of tau-microtubule binding reducing oligodendrocyte process number and length [129]. Expression of an inducible, truncated tau (which retains the Fyn-binding domain but lacks microtubule-binding capability) in oligodendrocytes induced demyelination and gait abnormalities in mice [160]. *MAPT*^*−/−*^ mice also show age-dependent degeneration of myelinated fibers, reduced nerve conduction, and progressive hypomyelination [157, 237], resulting in motor [157] and nociceptive [237] impairments. Tau knockdown also restricts recovery after sciatic nerve damage, with mice showing defective myelin debris clearance and severely impaired Schwann cell migration and differentiation [285]. Similarly, *MAPT*^*−/−*^ mice showed a worse clinical outcome after experimental autoimmune encephalomyelitis (EAE) (a model of demyelinating disease) [277]. Taken together, it seems likely that tau plays a key role in forming, maintaining, and repairing myelin, potentially via the regulation of microtubule dynamics [129]. Tau hyperphosphorylation may disrupt this key function in disease, contributing to the commonly observed white matter pathology. Whether myelination deficits will become a problem for tau-targeting therapeutics remains to be seen, but caution may be required to ensure normal oligodendrocyte and Schwann cell function is maintained.

### Aβ and its cleavage enzymes may regulate myelination

A recent study reports that low concentrations of Aβ oligomers can *enhance* oligodendrocyte survival in vitro [215]*.* Aβ peptides also induced translation of MBP, promoted oligodendrocyte differentiation, and improved remyelination in demyelinated cerebellar slices [215]. *BACE1*^*−/−*^ mice show delayed myelination and reduced myelin thickness in the PNS and CNS [110, 280], as well as impaired remyelination of peripheral nerves after injury [109, 111]. However, *BACE1*^−/−^ phenotypes may be Aβ-independent, relying instead on the cleavage of neuregulins [109], highlighting a potential off-target effect of lowering Aβ through BACE1 inhibition. Interestingly, BACE1, neuregulin-1, and Aβ expression increase during myelin repair following ischaemic stroke, suggesting a potential role in the myelin repair process [190]. However, oligomeric Aβ_1-42_ can also reduce oligodendrocyte number and induce motor deficits when injected intracerebroventricularly in mice [290]. Interestingly, a recent study has found that the secreted metalloprotease ADAMTS4, located exclusively in oligodendrocytes in adult mice, is responsible for the production of highly amyloidogenic, N-truncated, Aβ_4-x_ peptides [271]. Further studies will, therefore, be required to elucidate the role of Aβ peptides and their cleavage enzymes in oligodendrocytes, and to determine whether Aβ-targeting therapies will be beneficial or detrimental in resolving white matter pathology in disease. Once again, it seems likely that a balance between preventing toxicity and maintaining physiological functions will be required.

## Aβ regulates vasculature

### Angiogenesis and Aβ: a question of balance?

There is mounting evidence that Aβ plays important roles in regulating vasculature. Studies in post-mortem human brains have found evidence for increased angiogenesis in AD [21, 58], with one study finding that vascular density in the hippocampus positively correlated with Aβ load [58]. Conversely, other studies have reported *reduced* vascularisation in AD brains [76]. Studies in AD mice found evidence for a dual-staged response to rising Aβ, with young animals showing increased vascular density compared to wild-type littermates, with reversal of the trend in old mice [21]. In vitro*,* Aβ can inhibit endothelial cell capillary formation in a dose-dependent manner and stimulates capillary degeneration at high concentrations [201]. At low concentrations, however, Aβ has been found to promote endothelial cell proliferation [27], induce formation of capillary-like structures [27], and increase vessel density in brain slice cultures [64]. In vivo studies using chick [27] or zebrafish embryos [40] and adult zebrafish retinas [51] find that Aβ peptides increase capillary density and induce formation of sprouting tip cells. *BACE1*^−/−^ mice also show a significant reduction in retinal vascular density [38] and *APP-*deficient zebrafish, or zebrafish treated with a BACE1 inhibitor, have shorter hindbrain vessels with fewer cerebrovascular branches [163]. Crucially, application of human Aβ rescued the vascular phenotype in both *APP-*deficient and BACE1 inhibitor-treated zebrafish [163], highlighting a primary role of endogenous Aβ in maintaining normal capillary density.

Whilst low levels of Aβ seem important for maintaining physiological vascularisation, increasing levels of Aβ in early AD have been shown to promote *pathological angiogenesis*, resulting in excessive vascularisation and disturbance to normal blood flow [21, 58, 64]. Excessive cleavage of APP, or direct inhibitory action of Aβ, may overwhelm or inhibit the activity of γ-secretase, thus reducing its availability to cleave Notch proteins, known angiogenesis inhibitors [27, 64]. Indeed, γ-secretase inhibitor treatment increases angiogenesis and vascularisation in a range of model systems [40]. Lowering Aβ levels, through immunisation against Aβ [20] or BACE1 inhibition [64], restores normal vascular patterns in AD models. Indeed, a meta-analysis of clinical trials reports that people with AD receiving immunisations against Aβ_1-42,_ show a significant reduction of endothelial cells and vascular density [20]. In another example of hormesis, either too little or too much Aβ may result in pathological alterations to capillary density (Fig. 4). Carefully controlling Aβ concentration will be important to restore and maintain physiological angiogenesis in AD.

### Aβ as a “vascular plug”

Clinical trials targeting Aβ have unintentionally provided evidence for a further role of Aβ in the vascular system: repairing leaks in the blood–brain barrier (BBB) [30]. A side effect of a number of active or passive anti-Aβ immunotherapies has been the appearance of microhaemorrhages and brain oedema [203]. This pattern of pathology, visible by MRI, has been termed “Amyloid-Related Imaging Abnormalities” (ARIA) and has become synonymous with Aβ immunisation [240]. A meta-analysis of 14 Aβ-targeting clinical trials reported a fivefold increase in the incidence of ARIA in treated versus placebo AD groups, with many trials showing a dose-dependent effect and increased incidence in *APOE4* carriers [203]. Aβ immunisation in AD mouse models [24] and aged mouse lemurs [121] can also induce ARIA-like cerebral microbleeds.

At present, it is not fully understood why removal of Aβ causes haemorrhages. Deposition of amyloid in blood vessels [cerebral amyloid angiopathy (CAA)] is a common feature of AD [166]. One possibility is that sudden removal of amyloid from blood vessels could compromise vascular integrity, as amyloid may have completely replaced the vessel wall in some instances (grade 2 CAA) [166]. Alternatively, a number of groups have proposed a relationship between Aβ and blood clotting. Platelets are the primary source of Aβ within the blood [43] and Aβ can induce aggregation of platelets [69]. It has been postulated that Aβ could play a physiological role as a vascular sealant, which could become overactivated with age and disease [30]. The expression of APP and Aβ rises in response to haemorrhagic damage, such as induction of microhaemorrhages [80] and chronic hypertension [33]. *APP*^−/−^ or *BACE*^−/−^ mice also show increased mortality after ischaemic injury, with severe deficits in reactive blood flow compared to wild-type controls [133]. Taken together, these findings add weight to the hypothesis that Aβ could serve a crucial role in vascular responses to injury, potentially explaining the appearance of ARIA after Aβ-immunotherapy. Conversely, high levels of Aβ can increase BBB permeability, potentially via reducing levels of tight junction proteins [127]. Interestingly, increasing BBB permeability promoted clearance of Aβ from the brain and improved cognition in a mouse model of AD, indicative of a physiological negative feedback mechanism [127]. Once again, this demonstrates the delicate balance between Aβ acting to support physiological versus pathological processes that must be considered to design safe, effective therapeutics.

## Response to injury

### Tau and Aβ increase after injury: a protective or pathological response?

Increasing evidence demonstrates that the levels of both tau and Aβ proteins increase following stroke [190], spinal cord injury (SCI) [195, 212] and traumatic brain injury (TBI) [282]. TBI promotes tau aggregation and spreading [67], with levels of tau pathology detected via PET correlating with poor long-term neuropsychiatric outcomes [251]. Whilst increases in tau often correlate with poorer outcome after injury [251], increased soluble Aβ has been shown to correlate with improved neurological status in individuals with a TBI [29]. Similarly, the formation of Aβ deposits in demyelinating lesions in people with multiple sclerosis (MS) has been proposed as a potentially protective response to axonal injury [89]. This raises the question of whether increases in tau and Aβ after injury serve to promote recovery, or whether they are involved in a detrimental pathological cascade.

### Aβ may promote recovery after brain injury

Attempts to clarify the function of increased Aβ after injury have produced conflicting results and it is often difficult to separate specific effects of Aβ from functions of APP, which is known to be upregulated in axons after injury and may have protective functions [100]. Whilst some groups found that *BACE1* knockout or inhibition improves recovery after sciatic nerve injury [72] or TBI [153], others report that *BACE1*^−/−^ mice have impaired remyelination after sciatic nerve lesion [109, 111] and worse functional outcome after SCI [195] or TBI [168]. Interestingly, both *BACE1*^*−/−*^ and *APP*^*−/−*^ mice have an increased risk of mortality following cerebral ischaemia, indicating that this is likely a consequence of Aβ loss [133]. Mannix et al. found that following controlled cortical impact (CCI), functional outcome is worse in *BACE1*^*−/−*^ mice compared to wild-type mice [168]. A follow-up study demonstrated that motor function (but not spatial memory or histopathology) could be improved by administration of Aβ_1-40_ to *BACE1*^*−/−*^ mice after CCI, demonstrating a potential role for Aβ in recovery [167]. In the same study, administration of Aβ to injured wild-type animals *worsened* outcome, demonstrating a hormetic role for Aβ [167] (Fig. 4). Grant et al. also showed that peripheral administration of Aβ could reduce paralysis and demyelination in a mouse model of EAE, providing further evidence of a role for Aβ in promoting recovery after injury [89].

### The effect of tau depends on the type of injury induced

Studies exploring the role of tau after injury have also been contradictory. *MAPT*^*−/−*^ mice suffer worse functional outcome after sciatic nerve injury [285] and EAE [277], reminiscent of tau regulating physiological myelination (discussed earlier). Several studies, however, report that *MAPT*^*−/−*^ mice are resistant to some functional and cognitive deficits after TBI, although this depends on whether the injury was repetitive [45] and whether short-term or long-term outcomes were measured [256]. A study by Bi et al*.* found that following experimental stroke, *MAPT*^*−/−*^ mice were protected from neurological deficits and excitotoxic brain damage [19]. The authors suggested a mechanism for this effect whereby tau normally mediates excitotoxic Ras/ERK signalling by regulating SynGAP1 postsynaptic compartmentalisation [19]. Whilst further experiments are required in a range of injury models, it seems likely that the positive versus negative effects of targeting tau will depend largely on the type of damage induced and the normal recovery mechanisms required. An apparent role for tau in myelin repair may mean that individuals with AD who have a history of MS, for example, could be at risk of worse outcome after receiving tau-lowering therapies. Similarly, Aβ-lowering therapies may increase the risk of brain damage in individuals who go on to suffer a TBI, which may be a risk in individuals who frequently suffer falls [260]. Therefore, patient history could be key in assessing the risk for negative outcomes associated with comorbidities, prior to the administration of tau- or Aβ-targeting therapeutics.

## Mitochondria and oxidative stress

### Mitochondrial dysfunction is a key component of AD pathology

Mitochondrial dysfunction is an early and important pathogenic feature of AD [98], and both tau and Aβ have been shown to separately and synergistically affect mitochondrial function [66]. Whilst most studies have examined the pathological effects of tau and Aβ on mitochondria, there is some evidence that these proteins may also have physiological roles.

### Aβ and tau deposition: cause of, or response to, oxidative damage?

Many studies have found an association between mitochondrial Aβ deposition and oxidative stress [66, 193]. However, the directionality of the observed events, i.e., whether Aβ accumulation is a cause of, or response to, oxidative stress, has proven difficult to ascertain. Somewhat counterintuitively, whilst oxidative stress is an early feature of AD, oxidative damage decreases alongside increased Aβ deposition during AD progression [193]. Notably, neurons containing an NFT show a 40–56% decrease in oxidised nucleosides compared to non-tangle-bearing neurons [193], leading to the proposal that Aβ and tau may have antioxidant properties [235]. Interestingly, a gene called saitohin, located in the intron downstream of exon 9 within the *MAPT* gene, interacts with peroxiredoxin 6, an antioxidant enzyme that protects cells from oxidative damage [79]. Whilst further research is required to explore the interaction of saitohin with tau, it seems likely that gene-level changes to *MAPT* may also impact the function of saitohin. Therefore, caution may be required when utilising tau- or Aβ-targeting therapies, to avoid potential exacerbation of oxidative damage.

### Tau as a regulator of mitochondrial mobility and health?

Tau has also been proposed to regulate mitochondrial function. Sapir et al*.* found that tau knockdown reduced mitochondrial mobility and increased the number of abnormal mitochondria [227]. A recent study reports that tau localises to the outer mitochondrial membrane under physiological conditions, proposing a role for tau in regulating mitochondrial association, and calcium transfer, with the endoplasmic reticulum [48]. However, overexpression of tau can also impair mitochondrial transport [65] and mouse models of tauopathy demonstrate deficits in mitochondrial distribution [135]. These seemingly contradictory results require further exploration and it seems likely that tau expression levels, post-translational modifications, developmental stage, and potential compensation from other MAPs will all impact whether tau serves a physiological or pathological role at the mitochondria.

## Aβ may function as an antimicrobial peptide

### The antimicrobial protection/infection hypotheses of AD

In 2002, Robinson and Bishop proposed that Aβ is normally produced to “bind toxic solutes” with the formation of amyloid plaques being “an efficient means of presenting these toxins to phagocytes” [220]. Since then, a number of groups have built upon the idea of amyloidosis as an innate immune response and have proposed that increased Aβ deposition, caused by continuous activation of this pathway through recurrent, chronic infection, may lead to the development of AD [182].

### Aβ rises after infection and is associated with pathogens in the CNS

A number of studies have shown that infection with a range of pathogens, including Herpes simplex virus-1 (HSV-1), *Chlamydia pneumoniae* and *Borrelia burgdorferi,* can increase production and deposition of Aβ in vitro and in vivo*,* where microbial DNA can be found associated with amyloid plaques (reviewed in [87]). Interestingly, a post-mortem study of individuals who had died from HIV/AIDS found that over half of them possessed extensive accumulation of Aβ in the brain, despite their young age (average age of death was 43 years old) [90]. Interestingly, treatment of HSV-1-infected cell cultures with the antiviral agent Acyclovir lowers intracellular Aβ accumulation and normalises BACE1 expression, leading to the proposal that antiviral therapies may be beneficial for the treatment of AD [162]. It is important to note, however, that human studies linking infections and AD are mostly correlational in nature. Whilst there is considerable evidence for a role of neuroinflammation in the progression of AD [106], it is possible that the increase in microbes in the AD brain is a response to rather than a cause of AD pathology. Breakdown of the BBB is common during neurodegenerative disease [248], and this could permit invasion of microbes circulating in the periphery, especially viruses, such as HSV, that are endemic in the population [283].

### Aβ has antimicrobial activity

The increased expression of Aβ after infection is proposed to be an innate immune response, and there is evidence that Aβ can act as an antimicrobial peptide (AMP) (reviewed in [87]). Soscia et al*.* found that Aβ exhibits antimicrobial action of equal or greater potency than LL-37 (a human antimicrobial peptide) against eight common microbes [236]. Application of Aβ also decreases the infectivity of HSV-1 [162] in cell lines, and reduces the growth of *Candida albicans*, Gram-negative, and Gram-positive bacteria [138, 236] in culture. In vivo*,* Kumar et al*.* found that Aβ overexpression significantly increases survival in 5xFAD mice infected with *S. Typhimurium*, and in *C. elegans* infected with *C. albicans* and *S. Typhimurium* [138]. The authors suggest that Aβ acts as an AMP by agglutinating and trapping microbes via the binding of its heparin-binding domain to carbohydrates in microbe cell walls [138]. Alternatively, Aβ may form cation channels in cell membranes, inducing cell death via calcium dyshomeostasis [126]. Interestingly, *APP*^−/−^ mice show a trend for increased mortality after infection [138] and a common side effect of Aβ-targeting therapies in clinical trials has been increased incidence of infections [87], although BBB disruption caused by removal of vascular amyloid could also explain this increased vulnerability [240].

## Regulation of iron homeostasis

### Iron homeostasis may be disrupted in neurodegenerative disorders

A number of groups have postulated that iron dyshomeostasis could contribute to AD and other neurodegenerative diseases [140], with iron accumulation resulting in cellular oxidative damage, dysfunction, and death via ferroptosis, an iron-dependent form of regulated necrosis [140]. It remains unknown whether iron dyshomeostasis is a cause, or consequence, of neurodegenerative pathology, but there is evidence that tau and APP may play physiological roles in regulating cellular iron transport.

### Tau and APP play a role in physiological iron transport

Whilst the role of Aβ in regulating iron homeostasis is unclear, APP has been proposed to have iron-export ferroxidase activity and interacts with, and stabilises, a major iron transport protein, ferroportin [16, 63]. Indeed, *APP*^*−/−*^ neurons retain more iron [63] and *APP*^*−/−*^ mice show vulnerability to oxidative damage from dietary iron [63] and exhibit exaggerated age-dependent iron accumulation in the brain and liver [16]. Tau may also regulate iron transport, likely through mediating transport of APP to ferroportin [145]. Lei et al*.* found that *MAPT*^*−/−*^ mice exhibit age-dependent iron accumulation associated with neurodegeneration, cognitive deficits, and parkinsonian-like motor deficits [145]. These deficits were rescued by treatment of aged *MAPT*^*−/−*^ mice with the iron chelator clioquinol [144]. Interestingly, they also found that tau levels were reduced by ~ 40% in the substantia nigra of individuals with PD [145], and the authors hypothesise that loss of tau may contribute to the extensive iron accumulation reported in this brain region in PD. The same group reported that lithium-mediated reduction of tau in wild-type mice, or primary cortical neurons, results in iron accumulation in the brain and reduced cellular iron efflux [143]. Taken together, the effects of tau-lowering therapeutics on iron homeostasis should be carefully considered when targeting tau in AD. As iron chelation has been shown to be beneficial in a *MAPT*^*−/−*^ mouse model [144], potentially detrimental side effects may be avoided by a combinatorial approach targeting both tau and iron dyshomeostasis.

## The role of tau and Aβ in the nucleus

### Tau is located at the nucleus and interacts with oligonucleotides

Whilst the majority of tau protein is located in the cytoplasm, there is clear evidence for nuclear association of tau in cell lines [156, 165, 234], primary neurons [11], and in mouse [267] and human [68, 216] brain tissue. Nuclear tau is mostly unphosphorylated [246] and is often localised to the nucleolar border, co-localising with constitutive heterochromatin [169] and ribosomal DNA (rDNA) [226]. In vitro*,* tau readily binds RNA [31] and DNA [39, 275]. Tau binds the minor groove of DNA, in a manner analogous to histones, via its proline-rich domain and microtubule-binding domain R2 [39, 275]. Tau’s ability to bind nucleic acids is disrupted by hyperphosphorylation [161], raising the possibility that such changes during disease may result in loss of function.

### Tau protects DNA from damage

Neurons continuously face the harmful effects of oxidative stress and evidence points to a key role for tau in preventing DNA damage and promoting repair [267]. Cellular stress, induced by heat shock or reactive oxygen species (ROS), results in dephosphorylation of tau, translocation to the nucleus [246, 267] and increases the capacity for tau to bind DNA [246]. In vitro, tau protects DNA from thermal denaturation, DNAse digestion, and ROS-mediated damage [39, 275]. Hyperphosphorylation prevents tau from binding DNA, resulting in DNA breakage and deficient repair under experimental stress conditions [161]. Whilst primary neuronal cultures from wild-type mice are resistant to hyperthermic conditions, DNA breaks caused by extensive heat shock become evident in tau-deficient neurons, damage which can be rescued by overexpression of human tau targeted to the nucleus [246]. In vivo, *MAPT*^*−/−*^ mice show increased DNA fragmentation under physiological conditions and are highly susceptible to DNA breakage after hyperthermic stress [267]. *MAPT*^*−/−*^ mice also show delayed *repair* of double-strand breaks after heat shock which was especially evident in the CA1 hippocampal subfield [267]. Caution may, therefore, be required to ensure that tau-lowering therapies do not increase neuronal susceptibility to DNA damage.

### Tau maintains chromosomal stability

Cells from individuals with *MAPT* mutations consistently show chromosomal aberrations, including breakages, gaps, aneuploidies, and translocations [222]. Interestingly, knocking out one or both copies of tau in murine splenocytes resulted in a marked increase in aneuploidy, indicating that *MAPT* mutations could induce dysfunction via loss of function, potentially through disruption of the mitotic spindle [88]. In *MAPT*^*−/−*^ neurons, hallmarks of normal pericentromeric heterochromatin (clustering of H3K9me3 and HP1α) are also disrupted [165, 169], indicating a role for tau in maintaining nucleolar integrity. Pericentromeric chromatin was also found to be disrupted in AD post-mortem brain tissue, further supporting a role for tau loss of function in disease [169].

### Tau as a regulator of transcription

In addition to maintaining genomic integrity, tau may also regulate transcription of genes involved in neuronal function. A recent paper reported that shRNA knockdown of tau in neurons greatly reduced both mRNA and protein levels of VGLUT1 [234]. Overexpressing wild-type tau, or forcing tau translocation to the nucleus, resulted in increased VGLUT1 transcription [234]. Remarkably, this effect was abolished when attempted with P301L-mutant tau, indicating a potential loss-of-function effect of this FTD-associated mutation. Tau has also been implicated in *repressing* transcription of a number of genes implicated in neuronal function [17], with tau depletion, resulting in upregulation of proteins such as BAF-57 (involved in neuron-specific gene repression) [11]. Additionally, tau knockdown alters rDNA transcription, although whether tau normally promotes [226] or represses [165] this process, and the implications for neuronal function, is still under debate. Overall, tau plays many roles at the nucleus and there is evidence for disruption of these functions in disease. Preserving the physiological functions of tau at the nucleus may, therefore, provide therapeutic benefit in tauopathies.

### Aβ at the nucleus - a caution for tau-lowering therapeutics?

Whilst the APP-processing product AICD is frequently associated with the nucleus (reviewed in [47]), a nuclear role for Aβ has been less forthcoming. Exogenously applied, or transfected, Aβ can translocate to the nucleus in vitro and is found at the nucleus in adult wild-type or APP/PS1 mice [12]. Some studies have suggested that Aβ_1-42_ can regulate transcription, including upregulation of APP and insulin-like growth factor receptors [12, 13]. However, most studies focus on the pathological roles of Aβ in the nucleus including inducing DNA breaks [244] and increasing chromosome mis-segregation by disrupting physiological tau function [88]. The fact that Aβ induces DNA damage provides an interesting link between potential Aβ and tau pathology in disease. Perhaps, increasing Aβ-mediated DNA damage results in reactionary alterations to tau that become overwhelmed in time. With this in mind, tau-lowering therapeutics without additional targeting of Aβ may need to be carefully considered to prevent additional DNA damage resulting from the removal of physiological protection.

## Aβ and tau may function as tumour suppressors

### Do individuals with AD have reduced incidence of cancer?

An increasing number of studies have reported a striking inverse association between AD and many types of cancer (reviewed in [30]), with one study reporting that the risk of developing cancer was 50% lower in individuals with AD [188]. This effect is not thought to be simply due to individuals with cancer dying earlier in life, as there is no such inverse correlation with other age-related disorders such as vascular dementia [221]. Of note, a recent paper by Rossi et al. showed that individuals with FTD possessing an *MAPT* mutation have an *increased* risk of developing cancer [223]. Together, such findings warrant exploration of whether tau and Aβ could function physiologically as tumour suppressors.

### Aβ can act as a tumour suppressor

Aβ has been proposed to act as a tumour suppressor under a number of experimental conditions [30]. Studies demonstrate that Aβ can suppress tumour growth [201] and inhibit cancer cell proliferation [202], with Aβ dimers/trimers being stronger tumour inhibitors than pentadecamers [202]. AD transgenic mice overexpressing Aβ also demonstrate reduced growth rate of implanted glioma tumours, with the authors proposing that high levels of Aβ could inhibit neoangiogenesis within the tumour mass [200]. Brothers et al*.* suggest that Aβ could also indirectly suppress tumour formation through intercepting oncogenic viruses, or via scavenging free metal ions, restricting availability of micronutrients required for cell proliferation [30]. It is worth noting that the majority of studies investigating Aβ as a potential tumour suppressor have used supraphysiological concentrations in their models. Therefore, it is difficult to discern whether Aβ has a role in tumour suppression at physiological concentrations in humans. A recent study found significantly decreased *BACE1* expression in invasive ductal carcinoma, indicating that reduced levels of Aβ could potentially increase the proliferation rate of this aggressive tumour [284]. Counterintuitively, *increased* levels of APP have been found in a number of cancers, with worse outcomes associated with higher APP levels (reviewed in [198]). Further research is, therefore, required to elucidate the contribution of Aβ and its processing pathways to cancer pathogenesis and the potential impacts of Aβ-targeting therapies on cancer risk.

### Loss of tau function may increase tumour incidence

As microtubules are crucial components of the mitotic spindle and regulate the cell cycle [82], it seems plausible that tau could play a role in tumourigenesis. Gargini et al*.* showed that tau expression levels vary in different cancer types and high tau expression levels correlate with increased patient survival [82]. Another study found that *MAPT* knockdown enhanced cell growth and invasion and suggested that tau may be a tumour-suppressive protein in clear cell renal cell carcinoma [95]. The finding that *MAPT* mutations increase the risk of developing cancer suggests that tau mutations may result in loss of a tumour-suppressive function [223]. On the other hand, increased *MAPT* expression correlates with poor prognosis and taxane (a microtubule-stabilising drug) resistance in gastric cancer [180]. It appears that tau may have different effects depending on the cancer type being studied, highlighting the heterogeneous nature of cancer and the need for further research. As with Aβ, any potential for increased risk of cancer must be carefully assessed when designing tau-targeting therapeutics for AD.

## Glucose metabolism

### Dysregulation of insulin signalling in AD

Diabetes is a well-established risk factor for developing AD [152]. Insulin receptors are densely expressed in the hippocampus, frontal cortex, and entorhinal cortex [49], with insulin playing key roles in a number of crucial CNS processes such as synaptogenesis, synaptic remodelling [46], and regulation of memory [175]. The link between impaired insulin signalling and cognitive decline has been demonstrated in humans and animal models [25, 49]. Indeed, impaired brain glucose metabolism is an early characteristic of AD, with one study reporting that up to 81% of individuals with AD had either type 2 diabetes or impaired fasting glucose, an indicator of prediabetes [119]. Whilst direct connections between glucose metabolism, tau, and Aβ have been made, it is important to highlight that the association between AD and diabetes could be, at least in part, due to diabetes-related blood vessel damage given the strong epidemiological links between many vascular risk factors and AD [152, 176].

### Tau regulates normal glucose metabolism

Marciniak et al*.* reported that *MAPT*^*−/−*^ mice exhibit insulin resistance in the hippocampus, suggesting that tau plays a normal role in the cellular response to insulin, a function that may be lost in AD [170]. The authors propose a mechanism, whereby tau normally interacts with PTEN, a phosphatase that inhibits insulin signalling, reducing its activity [170]. Tau is expressed in the pancreas [296], and *MAPT*^*−/−*^ mice have been shown to develop pancreatic β cell dysfunction and glucose intolerance [279]. A recent imaging study in humans found that Aβ affected tau-glucose metabolism associations [2]. This study reported that during normal ageing, small increases in tau are associated with *increased* glucose metabolism, but large accumulations of hyperphosphorylated tau associated with Aβ deposition correlated instead with hypometabolism, suggestive of a hormetic function for tau (Fig. 4), or Aβ-mediated dysregulation of normal tau function [2]. Interestingly, data from genome-wide association studies have shown that the *MAPT* H1 haplotype is associated with increased glucose intolerance in humans [170]. Taken together, tau likely plays a role in the regulation of homeostatic glucose metabolism. How this changes during disease, for example by loss of function caused by Aβ accumulation, or the potential effects of tau-targeting therapies on insulin signalling, requires further exploration.

### Aβ-processing enzymes regulate insulin signalling

Whilst Aβ has been proposed to dysregulate insulin signalling [25], there is mounting evidence that its cleavage enzyme, BACE1, which is strongly expressed in the pancreas [298], plays crucial physiological roles in glucose homeostasis. Indeed, *BACE1*^*−/−*^ mice show impaired insulin expression in the pancreas [104] and siRNA-mediated BACE1 knockdown significantly reduces insulin mRNA and protein in insulinoma cells [104]. However, another study reports that *BACE1*^*−/−*^ mice have enhanced insulin sensitivity [177]. Notably, hyperglycaemia increases BACE1-mediated production of Aβ [142], indicative of a potentially vicious cycle between amyloid and glucose dysregulation. This also highlights the likely contribution of diseases such as diabetes mellitus to AD development and emphasises the potential of nutritional intervention strategies as a means of treating and preventing AD.

## Conclusion

**Fig. 5:**
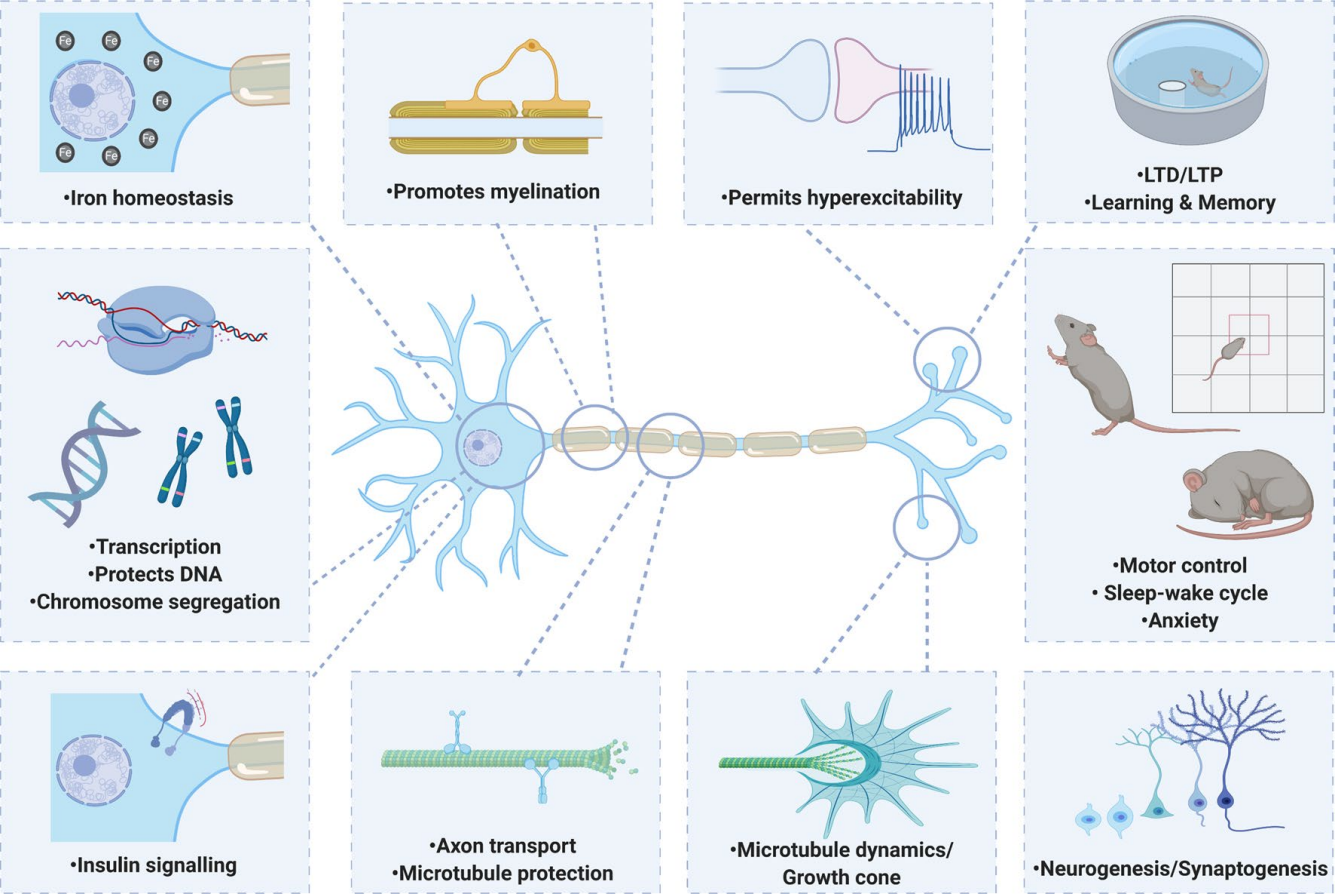
A schematic representation of the suggested physiological roles of tau in the brain and body. Created with https://biorender.com/

**Fig. 6:**
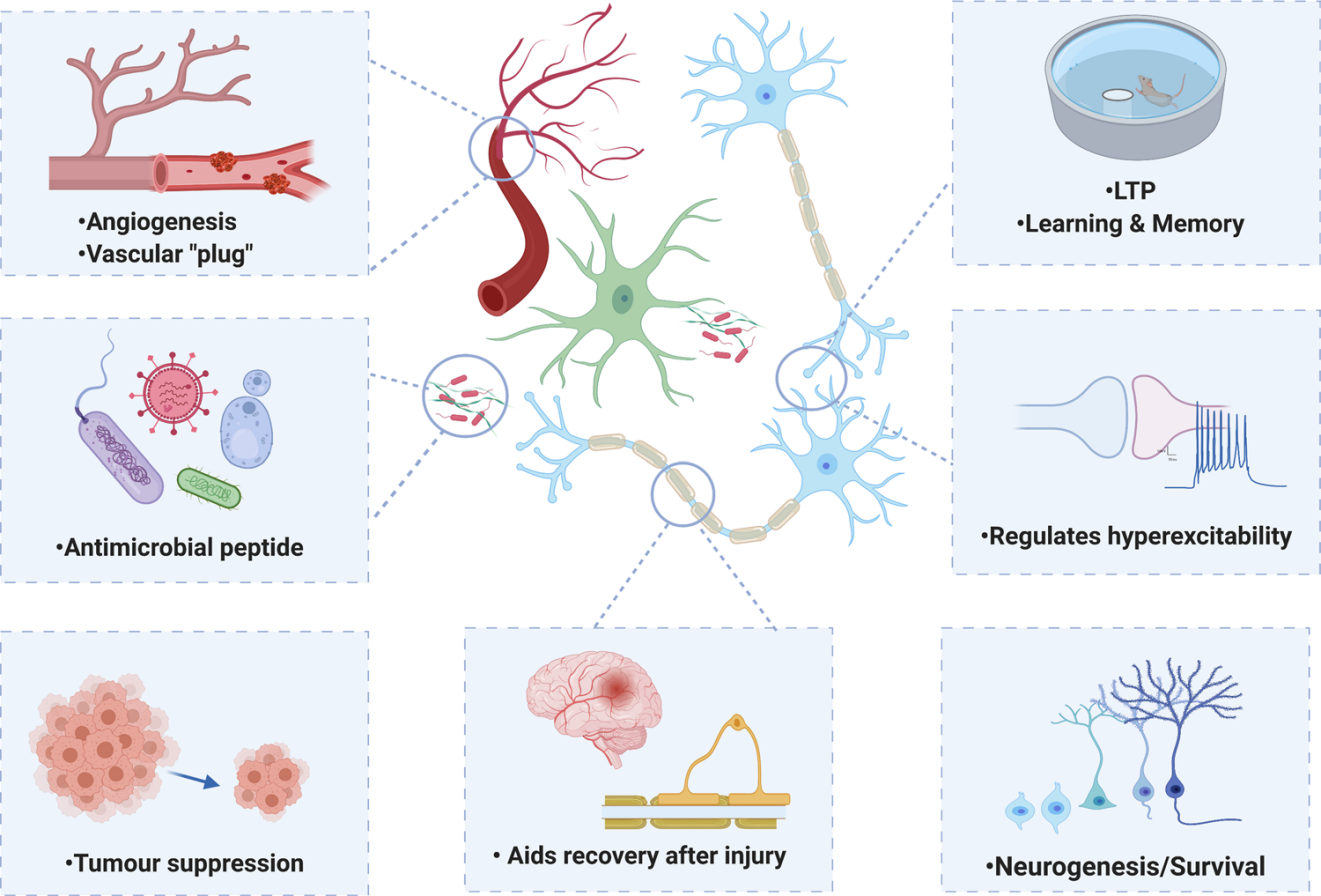
A schematic representation of the suggested physiological roles of Aβ in the brain and body. Created with https://biorender.com/

From molecular interactions with DNA to influencing complex behaviour, the physiological roles of tau (Fig. 5) and Aβ (Fig. 6) are multifaceted, dynamic, and at times, contradictory. This review emphasises the complexity of untangling physiology from pathology, as well as the wide-reaching implications, for both the brain and body, that can be induced by subtle protein alterations. Whilst the range of functions which we discuss are diverse, common themes emerge:

- The functions of tau and Aβ are influenced by their location. Shifts within the cell, expression in different cells or locations within the body, can alter the roles that these proteins play, whether they are beneficial or harmful, and how therapeutic treatments will influence function.
- Different isoforms, aggregation status, and post-translational modifications can dramatically alter the function of tau and Aβ.
- The concentrations of tau and Aβ are crucial for regulating physiological versus pathological function. This “hormetic nature”, where too much or too little protein causes functional deficits, raises the likelihood of a “therapeutic sweet spot” where the physiologically optimal concentrations lie, outside of which may result in damage (Fig. 4).
- AD is likely a combination of both gain- and loss-of-function phenotypes. Physiological responses can become hijacked to become toxic, detrimental functions can appear, or normal functions can be lost during disease-related changes.
- Contradictory findings are common in the literature. The potential for developmental compensation in constitutive knockouts, as well as experimental differences in factors such as age, sex, and environment, will all impact outcome. Future studies testing multiple timepoints, acute versus constitutive knockdown, different genetic backgrounds, mixed-sex cohorts, and increasing comparisons between animal and human tissue will greatly clarify genuine phenotypes from experimental artefacts.

With the above in mind, a meticulous approach to therapeutic development is crucial. Therapies must balance potentially detrimental effects of targeting tau (Table 1) and Aβ (Table 2) with the benefits of disrupting pathology. Targeting treatments early in the disease cascade, considering the individual’s medical history (such as previous injury and comorbidities), engaging with modifiable lifestyle risk factors and the use of combinatorial therapies will likely be important to obtain the best outcome for individuals. By understanding the functions of tau and Aβ in health, we can gain greater insight into their contribution to disease. Such research will be vital for the development of safe and effective therapeutic strategies.

## Abbreviations

Aβ: Amyloid beta
AD: Alzheimer’s disease
AICD: Aβ intracellular domain
AMP: Antimicrobial peptide
APP: Amyloid precursor protein
ARIA: Amyloid-related imaging abnormalities
ASD: Autism spectrum disorder
BACE1: Beta-site amyloid precursor protein cleaving enzyme 1
BBB: Blood–brain barrier
BDNF: Brain-derived neurotrophic factor
CAA: Cerebral amyloid angiopathy
CBD: Corticobasal degeneration
CCI: Controlled cortical impact
CNS: Central nervous system
CSF: Cerebrospinal fluid
EAE: Experimental autoimmune encephalomyelitis
FTD: Frontotemporal dementia
FTD-P17: Frontotemporal dementia with parkinsonism 17
HSV: Herpes simplex virus
iPSC: Induced pluripotent stem cell
LTD: Long-term depression
LTP: Long-term potentiation
MAP: Microtubule-associated protein
MAPT: Microtubule-associated protein tau
MBP: Myelin basic protein
MS: Multiple sclerosis
MWM: Morris Water Maze
NFTs: Neurofibrillary tangles
PD: Parkinson’s disease
PNS: Peripheral nervous system
PSD: Postsynaptic density
PS1: Presenilin-1
PSP: Progressive supranuclear palsy
rDNA: Ribosomal DNA
ROS: Reactive oxygen species
SCI: Spinal cord injury
TBI: Traumatic brain injury
TGN: Trans-Golgi network
TTLL6: Tubulin tyrosine ligase-like 6
3R: Tau with 3 microtubule-binding domains
4R: Tau with 4 microtubule-binding domains
